# Going the extra mile – creating a co-operative model for supporting patient and public involvement in research

**DOI:** 10.1186/s40900-016-0025-z

**Published:** 2016-04-04

**Authors:** Adele Horobin

**Affiliations:** 1 National Institute for Health Research (NIHR) Nottingham Hearing Biomedical Research Unit, 113 The Ropewalk, Nottingham, NG1 5DU UK; 2 Nottingham University Hospitals NHS Trust, Nottingham, UK

**Keywords:** ‘Going the Extra Mile’, Patient and public involvement, Share-bank, Time-bank, Collaborative, Regional, Cross-organisational

## Abstract

**Plain English summary:**

In 2014, the Chief Medical Officer and Director General of Research and Development commissioned a review of patient and public involvement in the National Institute for Health Research. The report on this review, entitled ‘Going the Extra Mile’ was published in March, 2015. It described the bold goal of expecting all people using health and social care, and increasing numbers of the public, to be aware of and choosing to be involved in research. This requires more effort to build public awareness of research and better support for the public and researchers to do patient and public involvement in research.

The author has created a new way of providing support for patient and public involvement based on co-operation between organisations. Termed ‘share-banking’, this model pools limited resources across organisations to deliver a regional programme of support activities for patient and public involvement over the long term. This includes helping organisations to share and learn from each other to avoid ‘re-inventing wheels’ (where separate organisations each develop the same thing from the beginning). The ‘Going the Extra Mile’ report recommends that local organisations should work together to deliver public involvement activities across a region. ‘Share-banking’ should help fulfil this recommendation.

**Abstract:**

The ‘Going the Extra Mile’ final report opened with the ambition to increase the public’s awareness, participation and involvement in research. It stated the need for public and researchers to be better supported to do public involvement. A new co-operative model, termed ‘share-banking’, has been developed whereby organisations pool limited resources to create and sustain support for patient and public involvement in research. This should fulfil the ‘Going the Extra Mile’ report’s recommendation to take a collaborative, cross-organisational and regional approach to public involvement.

## Letters

### Going the Extra Mile report

The ‘Going the Extra Mile’ final report and recommendations of the ‘Breaking Boundaries’ strategic review of public involvement in the National Institute for Health Research (NIHR) set out a clear goal [1]. It stated that “By 2025 we expect all people using health and social care, and increasing numbers of the public, to be aware of and choosing to contribute to research…” pp10. This is an ambitious target, and one which supports the Government’s mandate to NHS England to promote participation by NHS organisations and patients in research [2] and concurs with Part 5 of the Health and Social Care Act 2012 to drive patient involvement across the NHS [3]. This requires significant investment in raising the profile of research by increasing awareness of what research is and how it contributes to health and social care, and education on how the public can contribute to research through participation, involvement and engagement.

The report also concluded that “the public and researchers need to be better supported to do public involvement…” pp17. A collaborative approach is key to the report’s seventh recommendation that there should be more connectivity in offering this support. Here, the emphasis is on encouraging regional partnerships between organisations in the NIHR infrastructure and with organisations beyond NIHR boundaries. These partnerships would work to “identify cross-cutting activity in [patient and] public involvement (PPI) and develop joint plans and stable resourcing” [1] pp18.

### Developing a new model for regional support

From the author’s own experience, funding appears to be scarce for PPI initiatives which sit outside of individual research projects or organisations. Financial challenges limit the ability to pay for an outside organisation to deliver training and development in PPI. This prompted the author’s thinking on how we can develop and sustain a regional cross-organisational, collaborative model. This is where the idea of a PPI ‘share-bank’ was born. Although conceived independently, the model has parallels with the social community movement ‘time-banking’ [4]. The principle behind it is that individual organisations all have something of value to offer in terms of support for PPI. That is, to inform researchers on how to design and carry out research ‘with’ or ‘by’ members of the public rather than ‘to’, ‘about’ or ‘for’ them, as defined by INVOLVE [5]. Also, to inform the public on ways that they can contribute to the design and running of research. The innovation comes in sharing that support in a fair, balanced manner. As with ‘time-banking’, everyone shares the skills that they have in a reciprocal relationship, without monetary payments.

### East Midlands PPI share-bank

The NIHR Nottingham Hearing Biomedical Research Unit is leading on establishing the East Midlands PPI ‘share-bank’. This pilot programme is being co-developed and delivered with colleagues from the Nottingham University Hospitals NHS Trust Research and Innovation Services, NIHR Nottingham Digestive Diseases Biomedical Research Unit, NIHR East Midlands Collaboration for Leadership in Applied Health Research and Care and the NIHR East Midlands Research Design Service. The programme will run for six months initially before being evaluated and will consist of training sessions and workshops, for researchers and public to attend alongside one another. The aim is to inform researchers about PPI and provide the opportunity to meet with members of the public who have an interest in research, and to help the public realise the breadth of contribution that they can make. Initially, the support offered by the ‘share-bank’ will be made available to researchers and public affiliated to those member organisations listed. Upon completion and evaluation of the pilot, it is hoped that the programme will be expanded to include other organisations in the East Midlands. Particularly, we hope to include those groups who presently have very little resource for delivering support in PPI themselves.

### Fulfilling the goals of the Going the Extra Mile report

Widening access to PPI support is surely of necessity when considering how we fulfil the vision set out by the ‘Going the Extra Mile’ report [1]. The collaborative nature of the PPI ‘share-bank’ fulfils the objective of developing a cross-organisational, locally grown, regional approach to PPI, improving ‘connectivity’. In addition, our model will allow for stable resourcing, the central premise being that it would not cost much extra to deliver a resource to 20 individuals from several organisations as 5 individuals from one organisation. The model should also remove ‘reinventing of wheels’ where a similar resource is developed from scratch by different organisations.

### Incentives to work together

We look forward to reporting on the outcomes of this new venture in PPI support. One would like to conclude this letter with the question to policy makers: “How should organisations be better incentivised to work together to deliver something for the common good? Is it enough to articulate a recommendation that organisations within the NIHR should work together and build partnerships with organisations beyond the boundaries of NIHR [1] and/or should there be financial incentives to encourage cross-organisational working?”

## Abbreviations

NIHR: National Institute for Health Research
PPI: Patient and Public Involvement

## References

[1] NIHR. Going the extra mile: Improving the nation’s health and wellbeing through public involvement in research. www.nihr.ac.uk/documents/about-NIHR/NIHR-Publications/Extra%20Mile2.pdf. Accessed 17 September 2015.

[2] Department of Health. The Government’s mandate to NHS England for 2016–17. January 2016.

[3] Health and Social Care Act 2012. Available at: http://www.legislation.gov.uk/ukpga/2012/7/contents/enacted. Accessed 3 March 2016.

[4] Timebanking UK. www.timebanking.org/. Accessed 17 September 2015.

[5] INVOLVE. What is public involvement in research? 2016. http://www.invo.org.uk/find-out-more/what-is-public-involvement-in-research-2/. Accessed 1 February 2016.

